# Quantifying the Impact of Environment Loads on Displacements in a Suspension Bridge with a Data-Driven Approach

**DOI:** 10.3390/s24061877

**Published:** 2024-03-14

**Authors:** Jiaojiao Li, Xiaolin Meng, Liangliang Hu, Yan Bao

**Affiliations:** The Key Laboratory of Urban Security and Disaster Engineering of the Ministry of Education, Beijing University of Technology, Beijing 100124, China; ljj@emails.bjut.edu.cn (J.L.); huliangliang@emails.bjut.edu.cn (L.H.); baoy@bjut.edu.cn (Y.B.)

**Keywords:** structural health monitoring (SHM), environmental loads, extreme gradient boosting, temperature time-lag effect, variational mode decomposition, correlation analysis

## Abstract

Long-span bridges are susceptible to damage, aging, and deformation in harsh environments for a long time. Therefore, structural health monitoring (SHM) systems need to be used for reasonable monitoring and maintenance. Among various indicators, bridge displacement is a crucial parameter reflecting the bridge’s health condition. Due to the simultaneous bearing of multiple environmental loads on suspension bridges, determining the impact of different loads on displacement is beneficial for the better understanding of the health conditions of the bridges. Considering the fact that extreme gradient boosting (XGBoost) has higher prediction performance and robustness, the authors of this paper have developed a data-driven approach based on the XGBoost model to quantify the impact between different environmental loads and the displacement of a suspension bridge. Simultaneously, this study combined wavelet threshold (WT) denoising and the variational mode decomposition (VMD) method to conduct a modal decomposition of three-dimensional (3D) displacement, further investigating the interrelationships between different loads and bridge displacements. This model links wind speed, temperature, air pressure, and humidity with the 3D displacement response of the span using the bridge monitoring data provided by the GNSS and Earth Observation for Structural Health Monitoring (GeoSHM) system of the Forth Road Bridge (FRB) in the United Kingdom (UK), thus eliminating the temperature time-lag effect on displacement data. The effects of the different loads on the displacement are quantified individually with partial dependence plots (PDPs). Employing testing, it was found that the XGBoost model has a high predictive effect on the target variable of displacement. The analysis of quantification and correlation reveals that lateral displacement is primarily affected by same-direction wind, showing a clear positive correlation, and vertical displacement is mainly influenced by temperature and exhibits a negative correlation. Longitudinal displacement is jointly influenced by various environmental loads, showing a positive correlation with atmospheric pressure, temperature, and vertical wind and a negative correlation with longitudinal wind, lateral wind, and humidity. The results can guide bridge structural health monitoring in extreme weather to avoid accidents.

## 1. Introduction

Long-span suspension bridges are highly flexible and highly susceptible to extreme weather conditions, such as large temperature fluctuations, storms, and high humidity, which inevitably accelerate the aging, damage, and displacement process of structural elements. The rapid development and application of structural health monitoring (SHM) systems have made it possible to fully acquire data on the dynamic response and load excitation on bridges, including bridge displacement, which is a crucial indicator of the bridge’s overall health condition [1]. Various technological methods are currently employed for bridge displacement monitoring, including in-contact sensors monitoring, GNSS vibration displacement monitoring, and the application of remote sensing technologies. These techniques complement each other, collectively eliminating measurement limitations and weather interference and thereby enhancing the accuracy of bridge displacement monitoring. In-contact sensors monitoring commonly involves accelerometers, strain gauges, and displacement sensors directly on the bridge structure to measure its deformation and the forces acted on the structures [2]. In the field of GNSS monitoring and remote sensing technology, bridge images and three-dimensional (3D) displacement data are both obtained through multiple data sources. By applying GNSS technology [3], drone monitoring [4], MTInSAR technology [5], etc., remote monitoring of bridge displacement can finally be achieved. Through appropriate data analysis techniques, the impact proportions and patterns of various environmental loads on displacement can be precisely quantified. This will help ensure the bridge’s sustainability, availability, and safety by ensuring accurate operational maintenance and health assessment under various extreme weather conditions.

Previous research has effectively identified the impacts of individual environmental loads on bridge structures through the analysis of bridge monitoring data. By analyzing wind speed and bridge displacement response data collected from the GeoSHM system, Meng and Owen et al. discovered that wind load can cause a significant vibration response of long-span bridge structures [3,6]. Li et al. [7] evaluated the temperature effect of reinforced concrete bridge towers under extreme cold weather conditions, which intensifies the risk of bridge structure cracking. Koo et al. [8] found that the thermal expansion of the bridge deck, main cables, and additional cables caused by structural temperature was the main factor in the overall deformation of the Tamar bridge. Zeng et al. [9] found that under normal atmospheric pressure and low atmospheric pressure conditions, the proportion of pores in concrete increased significantly, and the compressive strength decreased significantly. The research above shows that environmental factors have an important impact on the safety and reliability of bridge structures. However, traditional approaches struggle to consider the complexity and uncertainty of multiple environmental factors, which makes it challenging to identify and utilize potential patterns and information in the data. 

With the increasing emphasis on the capabilities of machine learning (ML) in handling large amounts of data, its application in the field of civil engineering has become more widespread. To effectively utilize and mine information from bridge health monitoring data, data mining methods such as support vector machines (SVMs) [10], random forest (RF) [11,12], gradient boosting regression trees (GBRTs) [13], and extreme gradient boosting (XGBoost) [14] have been employed. The extensively employed RF algorithm, as applied by Sun [11] for forecasting the cumulative vertical displacement of bridge structures, still presents opportunities for accuracy enhancement. Among many such algorithms, the XGBoost algorithm distinguishes itself with its high accuracy and outstanding generalization, garnering favor from scholars. Liu et al. [15] utilized the XGBoost algorithm to establish a regression model that correlates the modal curvature variations of bridges with the degree of damage, thereby quantifying bridge damage. Xin et al. [16] employed the XGBoost and long short-term memory (LSTM) model for long-term traffic prediction of large-span cable-stayed bridges. These studies demonstrate that the XGBoost model is capable of processing and learning from bridge health monitoring data in the field of civil engineering while possessing strong predictive capabilities.

To further investigate the correlation between 3D displacements and environmental loads, the relevant components need to be extracted from the recorded dynamic responses. One of the most effective techniques employed for this purpose is variational mode decomposition (VMD), which effectively minimizes mode-mixing [17,18]. However, bridge sensors are susceptible to high-frequency noise interference beyond the target signal. To address the problem of VMD over-refining high-frequency noise, a combined approach with WT denoising is utilized to reduce high-frequency noise and enhance signal quality [19]. In this study, the fusion method has produced reliable results. Sun et al. [20] utilized a knock recognition method that combines wavelet transform and the VMD algorithm to eliminate interference noise components and extract knock characteristics. Wu et al. [21] proposed a high-voltage shunt reactor denoising method that combines improved VMD and WT that exhibits higher signal denoising capability and preserves more signal details.

The majority of recent studies on how environmental factors affect bridge deformation mainly focus on considering a single environmental variable. Moreover, the actual environment in which bridges are located is complex and ever-changing. Thus, this study proposes a data-driven method based on the XGBoost model, combined with WT denoising and VMD methods. This approach correlates wind speed, temperature, air pressure, and humidity with the three-dimensional displacement response of the bridge span and determines the influence of various environmental loads on the displacement of the bridge structure. With the GNSS and Earth Observation for Structural Health Monitoring (GeoSHM) system of the Forth Road Bridge (FRB) located in the UK, the approach considers the effects of temperature, wind, atmospheric pressure, and humidity loads on the 3D displacement of the bridge and eliminates the time-lag effect induced by temperature in the structural displacement data. The impact of various loads on displacement is quantified separately using partial dependence plots (PDPs). Additionally, correlation analyses are performed for the displacement components and loads. Finally, the importance of various environmental loads on the displacement is explained, and the impact trends of each feature on the prediction are also investigated.

Following this introduction, Section 2 provides a comprehensive review of the employed methodologies and outlines the proposed approaches. Section 3 describes the selected case study, an in-service road–rail suspension bridge, and details the source and processing of the dataset. In Section 4, the XGBoost model is employed to predict 3D displacement, and the importance indicators predicted by the model are globally and individually quantitatively explained. Time-domain denoising and modal decomposition are applied to displacement data, followed by correlation analysis between displacement components and actual environmental loads. Section 5 includes the conclusion regarding the work above.

## 2. Methodology

**Figure 1:**
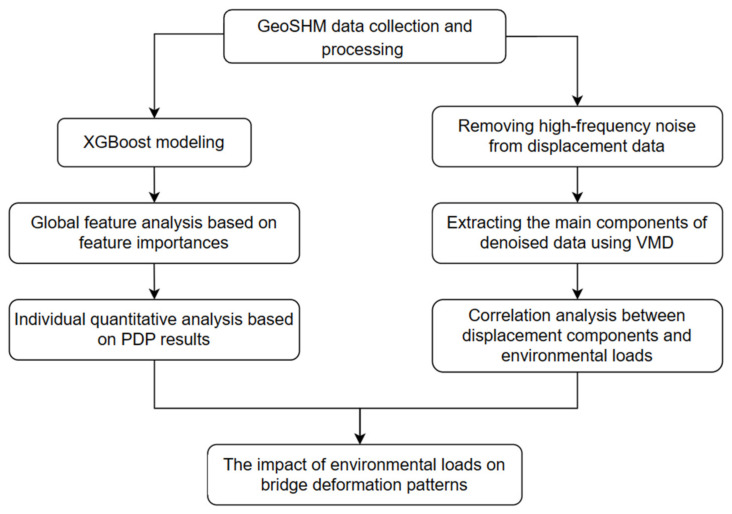
The main steps of the approach.

The main steps of the approach are shown in Figure 1. In the first part, the XGBoost model is established for displacement prediction based on the multi-source monitoring data of the GeoSHM system. A global analysis is conducted based on the importance of each feature in model training, with individual quantification and interpretation performed using PDPs. In the second part, the displacement data is denoised using WT, and the main components are extracted by processing the denoised data through VMD. These components are then correlated with environmental loads to validate and further explore the results obtained in the first part.

### 2.1. The XGBoost Model

XGBoost is a machine learning algorithm based on the gradient boosting tree model. It iteratively trains multiple weak learners and finally integrates a strong learner. This model can automatically learn the importance of features and select features based on the degree of contribution. It optimizes the objective function by introducing regularization terms and second-order Taylor expansion to improve calculation accuracy and effectively prevent overfitting [14].

The core idea of this algorithm is the boosting idea. In each iteration, a new decision tree is trained through fitting the gap between the actual value and the predicted value output by the previous round of the model. Then, the prediction results of all decision trees are added to obtain the final prediction model.

The algorithm formula is as follows:

(1) $${\hat{y}}_{i}^{k}=\sum_{k=1}^{k}f_{k}(x_{i})={\hat{y}}_{i}^{\left(k-1\right)}+f_{k}(x_{i})$$

In this equation, ${\hat{y}}_{i}^{k}$ is the prediction result of the sample model i after the *k*-th iteration; $f_{k}(x_{i})$ is the prediction result of the *k*-th decision tree model, where $x_{i}$ represents the feature vector of the *i*-th sample.

The objective function formula is updated with the following iteration:

(2) $$Obj^{\left(k\right)}=\sum_{i=1}^{n}l\left({\hat{y}}_{i},y_{i}\right)+\sum_{k=1}^{k}\Omega \left(f_{k}\right)$$

In this equation, Obj is the objective function to be minimized, representing the combined loss and regularization terms; $l\left({\hat{y}}_{i},y_{i}\right)$ is the loss function of the model; and $\Omega \left(f_{k}\right)$ is the regularization term controlling the complexity of the model, where $f_{k}$ represents the *k*-th decision tree model.

The regular items are split into the first *k* − 1 items and the *k*-th item. For the *k*-th tree, the first *k* − 1 trees have been trained, and the designed parameters and variables are recorded as constant *c*. Using Taylor’s formula to approximately expand the loss function, the objective function is obtained as follows:

(3) $$Obj^{\left(k\right)}=\sum_{i=1}^{n}\left[l\left(y_{i},{\hat{y}}_{i}^{\left(k-1\right)}\right)+g_{i}f_{k}\left(x_{i}\right)+\frac{1}{2}h_{i}f_{k}^{2}\left(x_{i}\right)\right]+\Omega \left(f_{k}\right)+c$$

where $g_{i}$ is the first-order derivative of the loss function; $h_{i}$ is the second derivative of the loss function; and c is a constant.

To explore the impact of environmental factors on displacement, environmental monitoring data were used as the input value of the model, and the displacement was used as the predicted value. The data were trained and tested based on the XGBoost algorithm, and a model for predicting displacement based on environmental loads was established.

### 2.2. Wavelet Threshold Denoising 

Displacement data collected by sensors inevitably contain high-frequency noise, such as electromagnetic noise. Therefore, these data should be preprocessed before further extracting the main components of displacement for analysis. To achieve this, this paper applies the WT denoising method to eliminate the contribution of high-frequency noise [22,23].

The WT denoising method is an algorithm based on multi-resolution analysis of wavelet transform. Its basic idea is that the wavelet decomposition coefficients of noise and signals in different frequency bands have different intensity distribution characteristics, and the signal is decomposed into different scales. For wavelet coefficients, noise is removed by setting thresholds, and then wavelet reconstruction is performed on the processed coefficients to obtain pure signals [24,25].

The soft threshold function expression is as follows:

(4) $$w_{\lambda }=\left\{\begin{array}{l} \left[\mathrm{sgn}(w)\right](\left|w\right|-\lambda )\;\left|w\right|\geq \lambda \\ 0\;\;\;\;\;\;\;\;\;\;\;\left|w\right|<\lambda \end{array}\right.$$

Among them, sgn is the sign function, used to determine the sign of real numbers. λ represents the threshold, determined based on the minimum mean squared error (MMSE) criterion; w represents the wavelet coefficient, and $w_{\lambda }$ represents the wavelet coefficient after assigning the threshold. 

The main steps of the WT denoising method include wavelet decomposition, threshold processing, and wavelet reconstruction. When performing wavelet decomposition on the original displacement signal $f_{0}$, Symlet-3 wavelet basis function is selected, and the number of wavelet decomposition levels is set to 4. The relationship between different levels is established by recursively applying the wavelet transform. Each level of decomposition generates approximation coefficients ($A_{i}$) and detail coefficients ($D_{i}$). $A_{i}$ contains the low-frequency components of the signal, while $D_{i}$ contains the high-frequency components of the signal, where high-frequency noise is typically manifested. By applying a soft thresholding process to the detail coefficients, smaller detail coefficients can be set to zero, effectively removing high-frequency noise. The approximation coefficients contain the main information of the signal, and by preserving them, the overall shape of the signal is retained. Through inverse wavelet transform, the signal f can be reconstructed based on the final approximation coefficients and detail coefficients, thereby eliminating high-frequency noise present in the signal acquisition and transmission process and improving the quality of the signal.

### 2.3. Variational Mode Decomposition (VMD)

VMD is a signal decomposition method that can decompose non-stationary and nonlinear signals into a series of intrinsic mode functions (IMFs). It overcomes the problems of endpoint effects and modal component aliasing in the empirical mode decomposition (EMD) method. It can reduce the non-stationarity of time series with high complexity and strong nonlinearity. The decomposition obtained contains multiple different frequency scales and is relatively stationary. The subsequence is suitable for non-stationary sequences. The core idea of VMD is to construct and solve variational problems [18].

(5) $$\left\{\begin{array}{l} \underset{\left\{\left.u_{k}\right\},\left\{\left.\omega_{k}\right\}\right.\right.}{\min}\left\{{\sum_{k}\left\|\partial_{t}\left[\left(\delta (t)+\frac{j}{\pi t}\right)\times u_{k}(t)\right]e^{-j\omega_{k}t}\right\|}_{2}^{2}\right\}\\ \;\;\;\;\;\;s.t.\sum_{k}u_{k}=f \end{array}\right.$$

In the equation, $\left\{u_{k}\}=\{u_{1},\ldots ,u_{k}\right\}$ and $\left\{\omega_{k}\}=\{\omega_{1},\ldots ,\omega_{k}\right\}$ represent the set of all IMF components and center frequencies, respectively; f is the decomposed signal; δ(t) is the Dirac delta function; and s.t. represents a constraint term.

To solve the constrained variational problem, quadratic penalty factor α and Lagrange multiplier λ(t) are introduced to transform the constrained variational problem into a non-constrained variational problem. The extended Lagrangian expression is

(6) $$L(\{u_{k}\},\{\omega_{k}\},\lambda )=\alpha {\sum_{k}\left\|\partial_{t}\left[\left(\delta (t)+\frac{j}{\pi t})\times u_{k}(t\right)\right]e^{-j\omega_{k}t}\right\|}_{2}^{2}+{\left\|f(t)-\sum_{k}u_{k}(t)\right\|}_{2}^{2}+\langle \lambda (t),f(t)-\sum_{k}u_{k}(t)\rangle$$

in which the penalty factor α represents the initial center constraint strength of each mode. The updates for $\mu_{K}^{n+1}$,$\omega_{K}^{n+1}$, and $\lambda^{n+1}$ are iteratively computed using the alternating direction algorithm to satisfy the conditions, as follows:

(7) $$\frac{\sum_{k}{\left\|{\hat{u}}_{k}^{n+1}-{\hat{u}}_{k}^{n}\right\|}_{2}^{2}}{{\left\|{\hat{u}}_{k}^{n}\right\|}_{2}^{2}}<\varepsilon$$

where ε is the discriminant accuracy. When the discrimination accuracy requirements are met, *k*-modal components are obtained, as indicated here:

(8) $$f={\sum_{i=1}^{k}imf\left(t\right)}_{i}$$

To better simulate periodic molecular systems, by using periodic boundary conditions, the original signal is copied to a certain length along the time axis and attached to both ends of the original signal, which is beneficial to removing endpoint effects. This paper uses VMD technology to separate the modal components from the displacement signal related to temperature, wind speed, etc. in the displacement to conduct correlation analysis [26].

### 2.4. Analysis Framework

**Figure 2:**
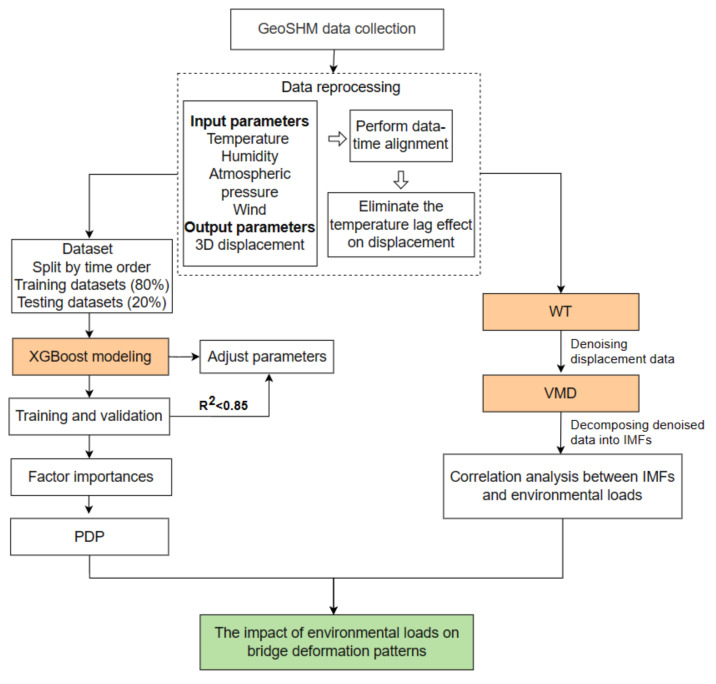
The framework of the data-driven approach.

The analysis framework is shown in Figure 2. Firstly, data are collected and processed from the bridge’s GeoSHM system to identify and eliminate the time-lag effect between temperature and displacement. Furthermore, environmental monitoring data are used as the input value of environmental load, and 3D displacement data are used as the output value. The datasets are divided into training and testing sets in a ratio of 0.8 and 0.2, and an XGBoost model is established to learn and predict displacement. When R^2^ > 0.85, model training reaches the best effect. The importance of all factors is extracted and PDPs are used to further visualize the relationships between all the data. Eventually, the WT denoising method and VMD methods are used to analyze the correlation between the obtained modal components and various environmental loads on the 3D displacement data. By using the methods above, the influence of environmental loads on bridge displacement can be determined.

## 3. The Forth Road Bridge and Dataset

### 3.1. The Forth Road Bridge and the GeoSHM System

**Figure 3:**
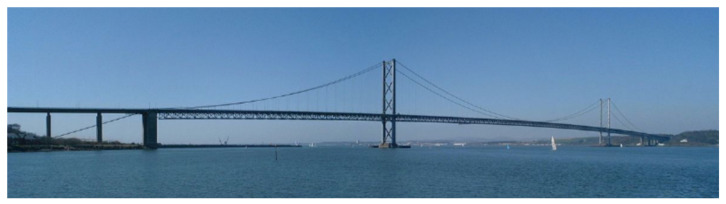
Forth Road Bridge.

The FRB is a major suspension bridge spanning the Firth of Forth near Edinburgh, Scotland, as shown in Figure 3. The bridge was opened to traffic in 1964 and has a main span length of 1006 m, with side spans measuring 408 m, making it one of the largest suspension bridges in the world in the 1960s. The main towers of the bridge reach a height of 150 m, and the main cables have a length of 2116 m. The bridge deck is composed of steel orthotropic plates, with two pedestrian walkways located on the outer sides of the main girder. Longitudinal gaps are incorporated between the walkways and the carriageway to enhance the aerodynamic stability of the bridge.

During certain extreme weather conditions, especially during storms, the FRB has been closed due to significant wind-induced reactions and high threats to vehicles and the public. Table 1 provides the restrictions imposed on the FRB under different wind speed conditions to ensure the safety of the structure. When the wind speed exceeds 64 mph (equivalent to approximately 29 m/s), all traffic is closed. It is estimated that the cost of closing one lane per day exceeds GBP 650,000. The maintenance of the bridge primarily relies on manual inspections.

To have a timely and comprehensive understanding of the bridge structure and operational condition, the GeoSHM project was initiated in 2014, funded by the European Space Agency, and jointly led by UbiPOS Ltd. and the University of Nottingham. The GeoSHM project integrates global navigation satellite systems (GNSSs) and earth observation techniques for the SHM of large-span bridges. The monitoring data provided by the GeoSHM project has been utilized by bridge management personnel to make decisions regarding the safety, operation, and maintenance of the bridge. Feasibility studies of GeoSHM have demonstrated that even small monitoring systems can provide a comprehensive understanding of the loading and response effects on the FRB, as well as identify abnormal deformations under extreme weather conditions [3,27].

**Figure 4:**
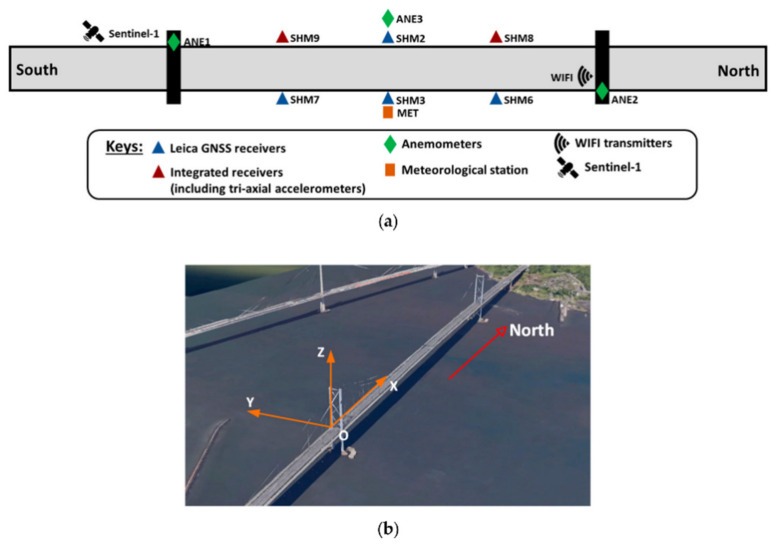
(**a**) The GeoSHM multi-source sensor system; (**b**) the bridge coordinate system (BCS) of the FRB.

Figure 4 depicts the distribution of the GeoSHM sensor system installed on the FRB. Three pairs of GNSS receivers are arranged on the main span of the FRB. The locations are, respectively, 1/4 span, mid-span, and 3/4 span. Real-time kinematic (RTK) GNSS positioning technology is used for processing GNSS measurements. GNSS receivers on the west side at 1/4 and 3/4 of the main span are integrated with three-axis accelerometers. Anemometers installed at the mid-span of the bridge and on top of the two main towers facilitate detailed correlation studies of wind loads on the FRB. In addition, a meteorological (MET) station is placed at the mid-span of the bridge to measure other environmental conditions, including temperature, atmospheric pressure, and air humidity. Data from the GeoSHM sensor system are collected at predefined sampling frequencies and transferred to the GeoSHM main server for processing analysis and storage using a combination of fiber optics, wireless networks, and the Internet [27,28].

### 3.2. Dataset

**Figure 5:**
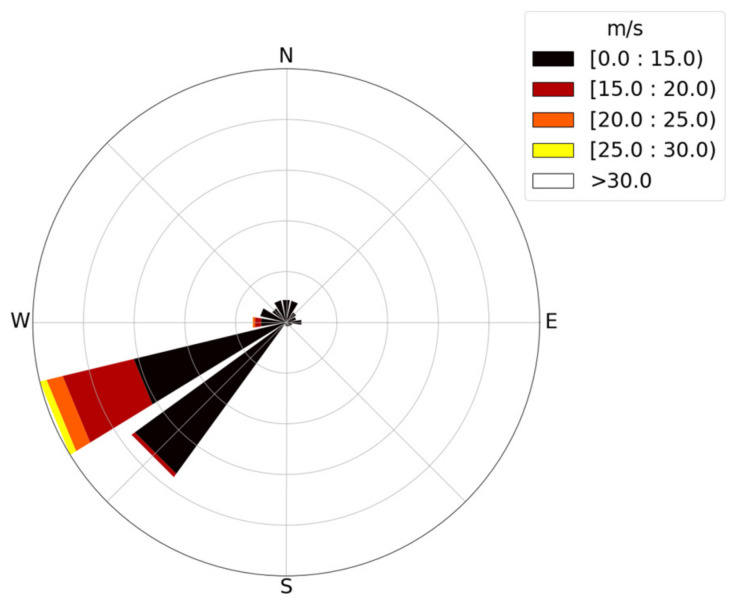
Wind rose for the FRB in February.

The dataset was collected in February 2023. The data include the bridge’s 3D displacement response and environmental monitoring data (wind speed, temperature, air pressure, and humidity). It should be noted that the displacements at mid-span SHM2 of the bridge in the X, Y, and Z directions in this paper correspond to longitudinal displacement, lateral displacement, and vertical displacement of the BCS, respectively. The data are obtained from the GNSS receiver and weather station at the mid-span position and the anemometer (ANE2) on the main tower on the north side. The GNSS receivers are Leica’s GS10 and DM3 receivers (Leica/UbiPOS, London, UK/Wetzlar, Germany). The manufacturer of the anemometer is Gill (Lymington, UK). All data are resampled every 10 min from the original datasets. The wind rose chart of the February data is shown in Figure 5. The offshore wind speed in this area is mainly distributed in the range of 0–30 m/s. Winds in the southwest direction have higher relative speeds and frequencies, while winds in other directions have lower speeds and lower frequencies. In particular, during storm Otto on 17 February, the FRB was hit by extremely strong winds. The maximum wind speed was close to 26.82 m/s, and its wind direction was mainly concentrated in the southwest.

Bridge components absorb or release heat through thermal conduction when the temperature changes, resulting in a lag effect in deformation. Therefore, this lag effect must be eliminated when conducting an in-depth study of the relationship between temperature and bridge deformation [22,29,30,31].

**Figure 6:**
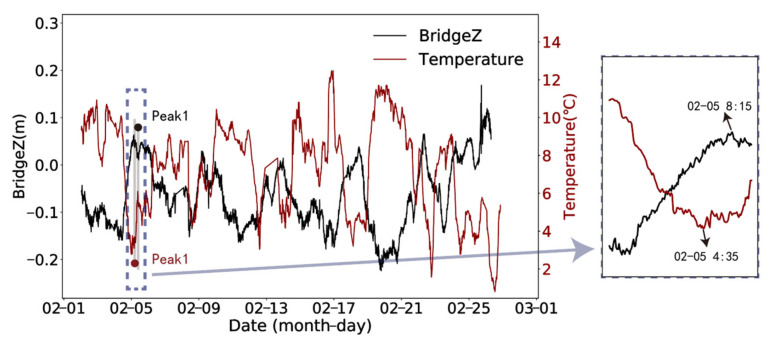
Time history curves of temperature and vertical displacement.

This paper takes vertical deformation as an example. Figure 6 shows that there is a certain time lag in the peaks of the deformation and temperature series. Consequently, it is vital to consider removing the hysteresis effect to more accurately determine the relationship between the two and properly assess the effects of temperature variations on the bridge.

The mathematical model for eliminating the time-lag effect between temperature and displacement can be defined as follows:

T(t) represents the temperature series, and D(t) represents the deformation series, where t is the time index. To eliminate the time-lag effect, a shifting function, Shift(T,τ), is employed to shift the temperature series T(t) by a certain time lag, τ, precisely aligning it with the displacement series D(t). Mathematically, this shifting function can be expressed as

(9) Shift(T,τ)=T(t−τ)

where τ is the lag time to be determined.

Once the temperature series is appropriately shifted, the Pearson correlation coefficient ρ between the shifted temperature time series and the displacement time series is calculated. The objective is to find the optimal lag time τ that maximizes the correlation coefficient ρ. The formula is as follows:

(10) $$\rho (\tau )=\frac{\mathrm{cov}(D(t),Shift(T,\tau ))}{\sigma_{D}\cdot \sigma_{Shift(T,\tau )}}$$

where cov denotes covariance; $\sigma_{D}$ and $\sigma_{Shift(T,\tau )}$ are the standard deviations of the displacement time series and the shifted temperature time series, respectively.

By optimizing the lag time τ to maximize the correlation coefficient ρ(τ), the time-lag effect between temperature and displacement can be effectively eliminated, ensuring accurate alignment and analysis of the two datasets.

**Figure 7:**
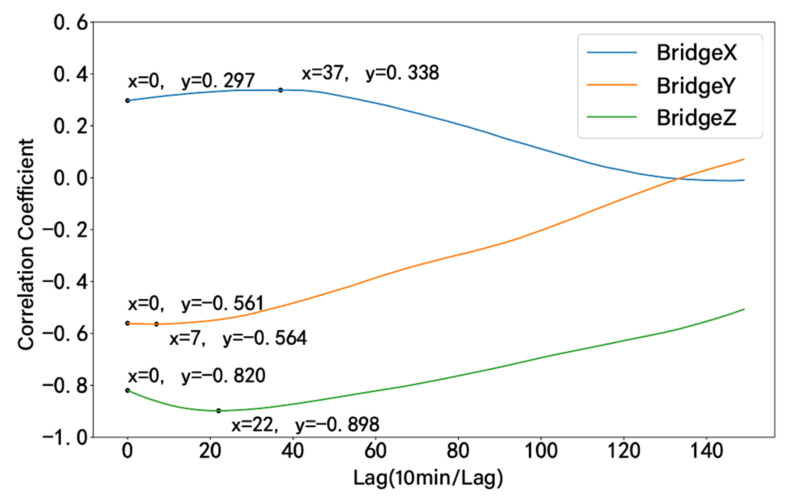
Hysteresis plot between temperature and displacement.

**Figure 8:**
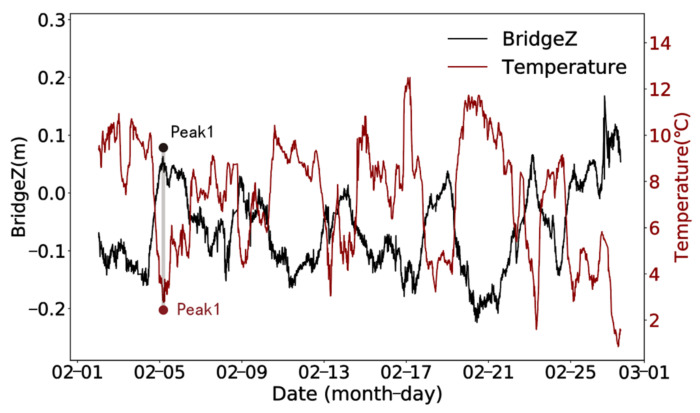
Time history curves of temperature and vertical displacement by eliminating the time-lag effect.

According to the time-lag results in Figure 7, it can be observed that the displacement data in different directions show different trends with temperature changes. The time lag of temperature on the vertical deformation of the bridge is the most obvious. The lag time is about 220 min. The absolute value of the correlation coefficient increases from 0.820 to 0.898, and the change amplitude is larger than the other two directions. Figure 8 presents the results of eliminating the time-lag effect of temperature on vertical displacement.

## 4. Results and Discussion

### 4.1. Quantification of Load–Displacement Impact

To ensure the reliability of the results, the training and testing of the XGBoost model are based on strict data preprocessing and ten-fold cross-validation methods, thus avoiding problems such as overfitting and the insufficient generalization ability of the model [11]. In this model, the displacements in the X, Y, and Z directions are used as target variables and are predicted based on the input load variables. The training and testing datasets account for 80% and 20% of the total datasets, respectively. The R^2^ values of the model for the three target variables on the test set are 0.885, 0.977, and 0.977, indicating a good fit between the predicted results of the model and the true values, with high accuracy and reliability.

**Figure 9:**
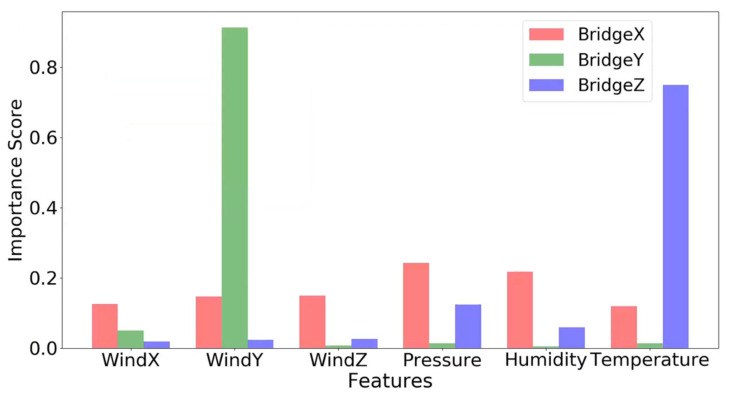
Feature importance plot obtained by the XGBoost model.

The feature importance of the XGBoost model refers to the contribution of each individual feature to the final prediction result during the model training process. This contribution is usually measured by evaluating the impact of features on model performance in the training data set, and this impact is often referred to as “split gain”. Features with higher feature importance play a more significant role in the prediction results of the model. In this paper, it can be used to evaluate the effect of environmental loads on displacement changes. Figure 9 shows the importance of different features when predicting bridge displacement using the XGBoost model.

Table 2 presents the importance scores derived from assessing the contributions of various environmental load factors to bridge displacements in the X, Y, and Z directions. It is evident that lateral wind contributes the most to the lateral displacement of the bridge, temperature contributes the most to the vertical displacement of the bridge, and the longitudinal displacement of the bridge is affected by multiple factors. 

For the X-direction displacement, atmospheric pressure has the highest importance score of 0.24. Other factors also cause similar degrees of deformation, and they all belong to the second most important contribution. This shows that the longitudinal direction of the bridge releases energy through expansion and contraction when facing the external environmental loads, thereby adjusting the structural state and producing deformation. For the Y-direction displacement, Y-direction wind has the highest importance score of 0.91, while scores for other factors are notably lower, all below 0.05. This shows that the structural form of the suspension bridge has high flexibility in its lateral direction, which makes the wind influence in the Y direction more significant. For the Z-direction displacement, temperature has the highest importance score of 0.75. This is because the materials in the bridge structure have relatively sensitive response characteristics to temperature changes. This is followed by atmospheric pressure and humidity, with scores of 0.12 and 0.06, respectively.

Based on the prediction results obtained by the XGBoost model, PDPs are introduced to individually quantify the impact of loads on the prediction results. By controlling for other characteristic factors such as the observed values, PDPs can evaluate the impact of specific characteristic variables on the model’s prediction output [32,33]. It can solve the problem of the feature importance obtained by model training not reflecting the positive and negative relationships precisely.

**Figure 10:**
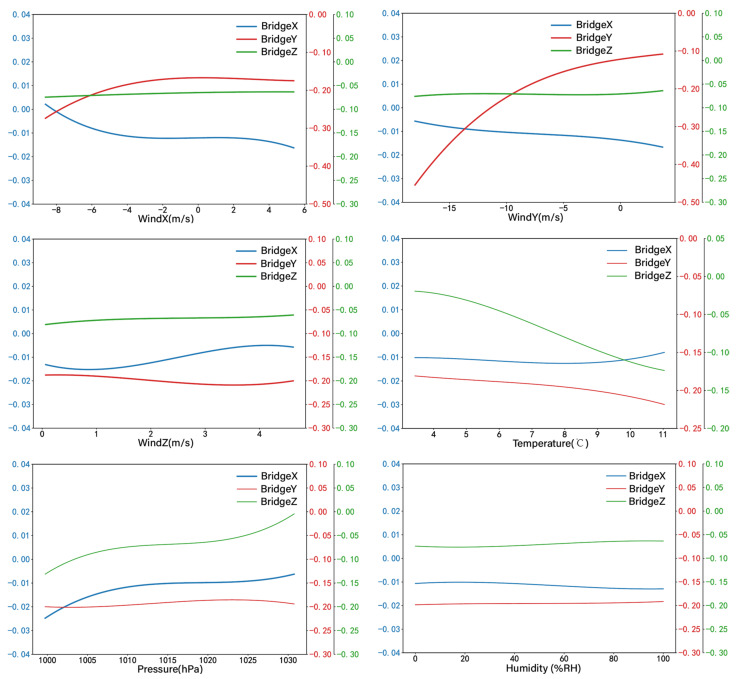
Partial dependency graph (PDPs, vertical axis unit: m).

According to Figure 10, the quantified relationship between bridge displacement and various factors can be observed. The X-direction displacement is influenced by multiple factors. As the atmospheric pressure increases from 1000 hPa to 1030 hPa, the displacement change gradually decreases, and the wind speed in each direction also has a certain influence on the displacement. The displacement change trend caused by temperature and humidity changes is relatively small. The Y-direction displacement is dominated by the variation of wind speed in the same direction. When the wind speed decreases from −40 m/s to −10 m/s, the displacement decreases from 0.45 m to 0.10 m. The relationship between the two components in the figure shows a clear positive correlation, whilst the influence of other factors on the displacement is not significant. The Z-direction displacement is mainly affected by temperature changes. When the temperature increases from 4 °C to 11 °C, the displacement decreases from −0.02 m to −0.125 m, and the relationship between the two shows a significant negative correlation. The influence of other factors on displacement is not significant. 

Furthermore, the trends in the Y- and Z-direction displacements concerning wind speed and temperature are opposite to those in the X-direction. These trends reveal the structural relationship faced by suspension bridges at mid-span, where a certain shrinkage of the materials in the bridge structure occurs in the longitudinal direction perpendicular to the direction of tension.

### 4.2. Correlation between Environmental Loads and Displacement

To validate the previous results and deeply analyze the components of bridge displacement related to various environmental loads, the displacement components are obtained by using WT denoising and VMD processing on the displacement data, and a correlation analysis is performed on this basis. 

#### 4.2.1. Temperature and Vertical Displacement

When the temperature rises, the suspension rods and main cables of the suspension bridge become longer due to thermal expansion, causing the suspension bridge to bend downward and the vertical displacement of the bridge to increase, which may negatively affect the stability and safety performance of the suspension bridge.

**Figure 11:**
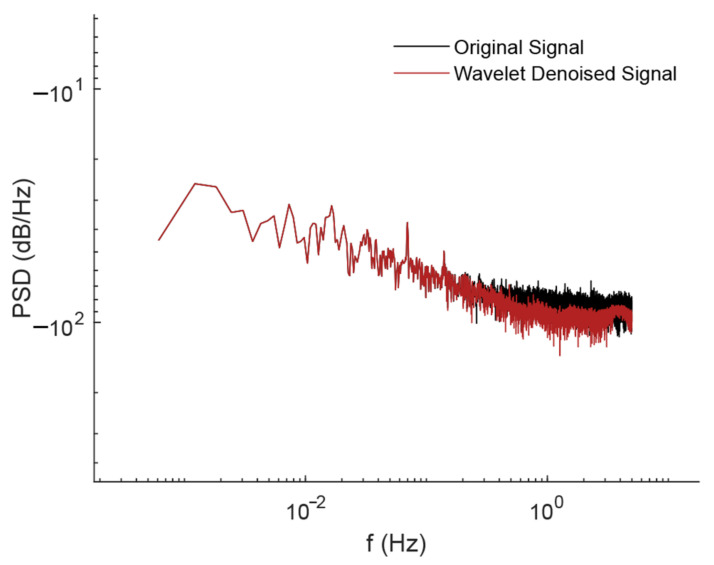
The power spectrum of vertical displacement before and after denoising.

The temperature and vertical displacement data are processed and analyzed in this paper to determine the link between the two. First, the displacement data are processed using the WT denoising method to reduce the noise interference on the analysis results. The power spectrum before and after denoising the displacement data is shown in Figure 11. When comparing them, a clear difference can be observed. After denoising processing, the low-frequency band signals are well preserved, which shows that the signals related to the vibration of the bridge itself are preserved as much as possible, while the higher frequency bands present clearer features.

**Figure 12:**
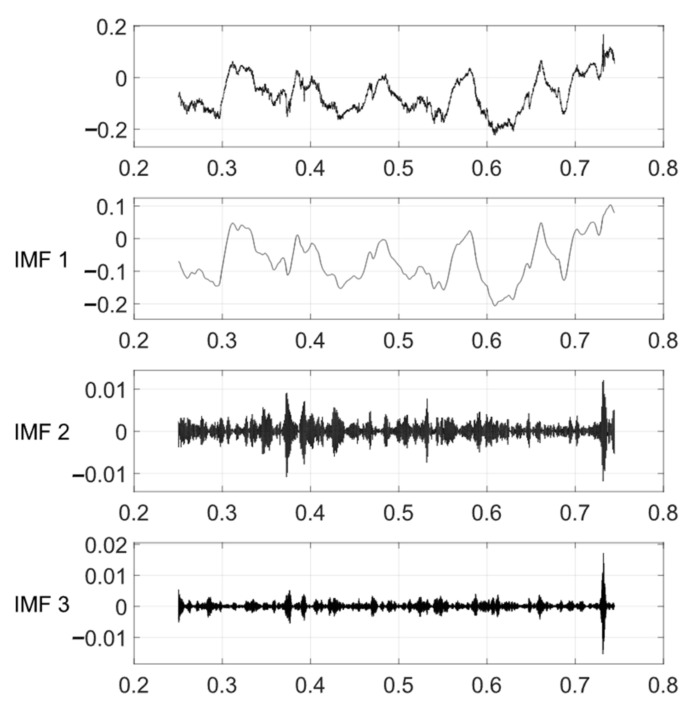
VMD of vertical displacement.

**Figure 13:**
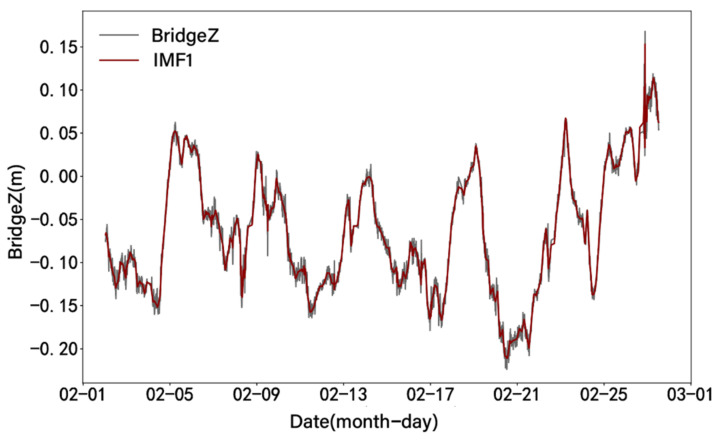
Comparison of denoised vertical displacement and IMF1.

**Figure 14:**
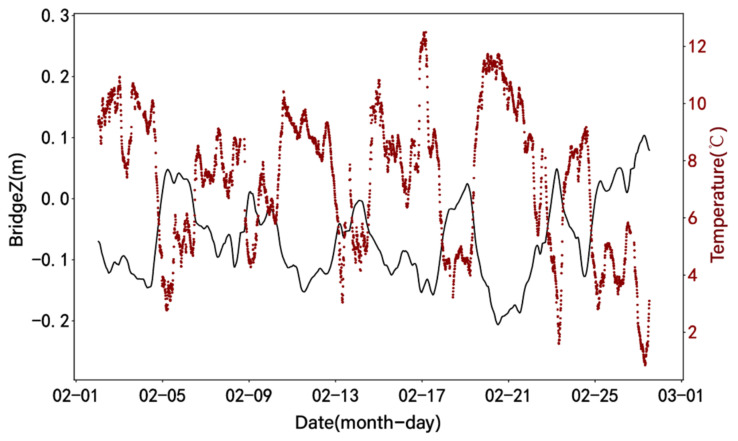
Time history curve of bridge Z (IMF1) and temperature.

The VMD method is used to decompose the noise-reduced displacement signal into three modal components ranging from low frequency to high frequency. After removing the additional signals at both ends of the signal, the VMD results of the vertical displacement are shown in Figure 12. By plotting the relationship between the denoised displacement data and IMF1 after displacement decomposition (Figure 13), it can be observed that IMF1 is highly consistent with the displacement trend. A comparative analysis shows that the changing trend of IMF1 has an obvious opposite relationship with temperature, as shown in Figure 14. The result obtained by calculating the Pearson correlation coefficient is −0.903, indicating that there is a significant negative correlation between IMF1 and temperature. This means that with an increase or decrease in temperature, the magnitude of IMF1 decreases or increases, indicating a decrease or increase in the vertical displacement of the bridge.

#### 4.2.2. Wind Speed and Lateral Displacement

When analyzing the lateral displacement of the FRB under strong wind conditions, it is important to understand the relationship between wind speed and lateral displacement. Based on historical data, the wind in February at the FRB is predominantly from the southwest direction. When this lateral wind acts on the bridge, it exerts a transverse force on the side of the bridge. This transverse force can have an impact on the bridge structure, especially when it exceeds the lateral resistance capacity of the bridge, resulting in lateral displacement. Such lateral displacement can affect the stability and safety performance of the bridge, particularly under strong wind conditions.

**Figure 15:**
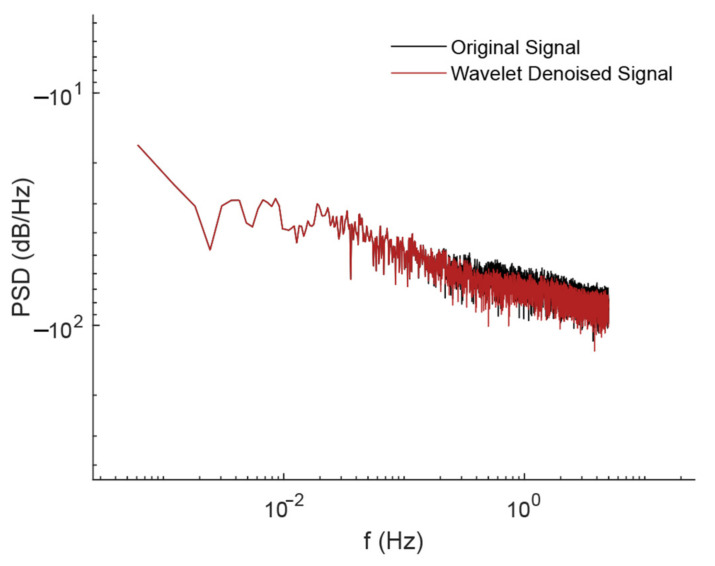
Power spectrum of lateral displacement before and after denoising.

**Figure 16:**
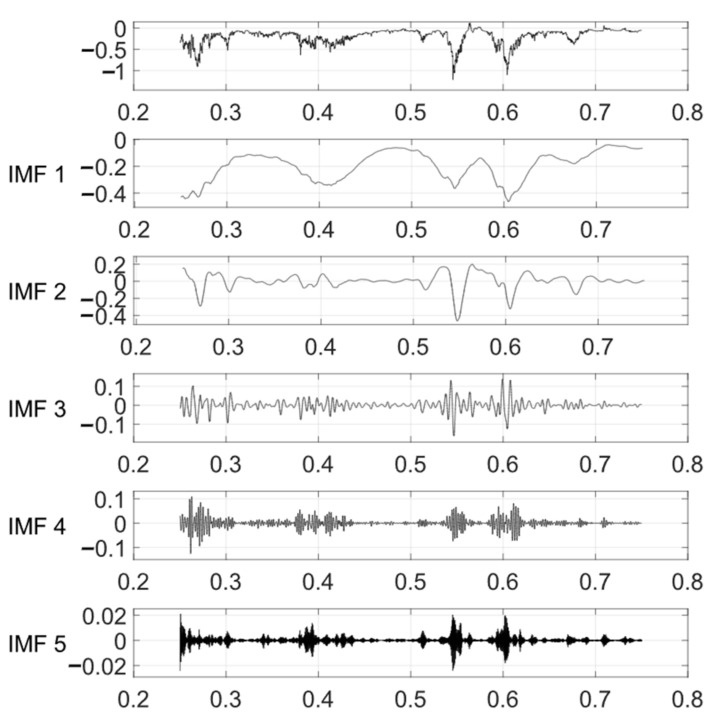
VMD of lateral displacement.

To gain a better understanding of the lateral displacement, the power spectrum before and after denoising the displacement data is shown in Figure 15. The VMD algorithm is used to decompose the denoised displacement data into different frequency components, as shown in Figure 16.

**Figure 17:**
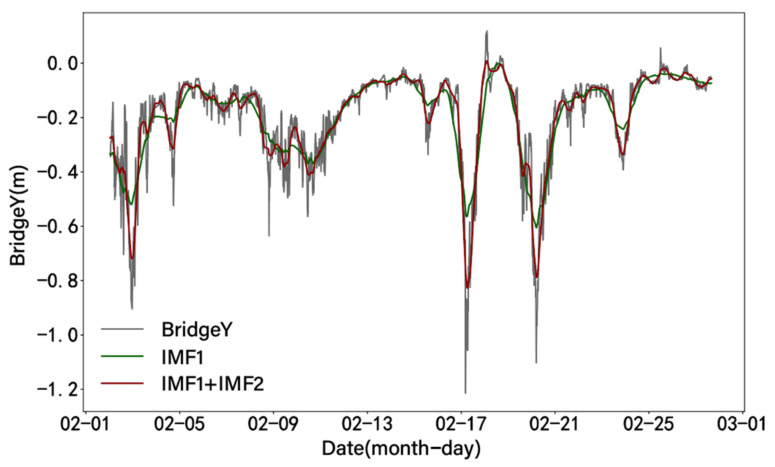
Comparison of denoised lateral displacement, IMF1, and IMF1+IMF2.

**Figure 18:**
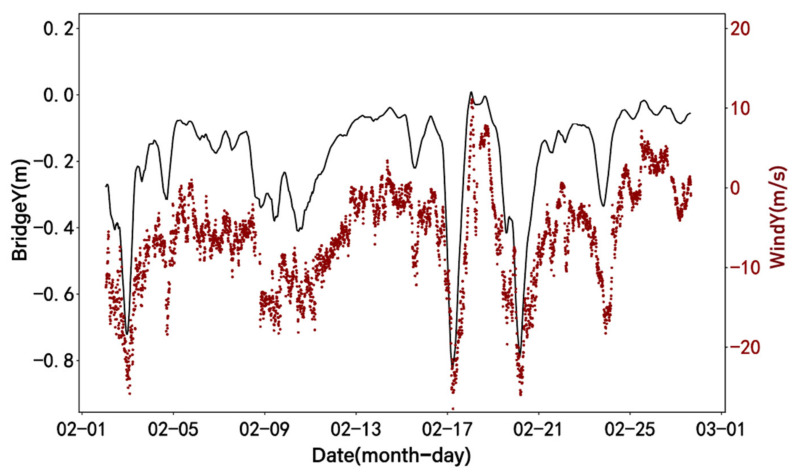
Time history curve of bridge Y (IMF1+IMF2) and wind speed.

By observing the waveform characteristics of IMF1 and IMF2, it can be observed that they are similar to the trend of the displacement data. By summing IMF1 and IMF2 and plotting the comparison with the denoised displacement data and IMF1, the result is shown in Figure 17. Encouragingly, the summed results are highly consistent with the general trend of the raw displacement data. For further analysis, the summed result of IMF1 and IMF2 is compared with the time series plot of wind speed, as shown in Figure 18. The results indicate a high degree of correlation between the two, with changes in wind speed corresponding to changes in lateral displacement. The calculated Pearson correlation coefficient is 0.896, indicating a strong positive correlation between lateral displacement and wind speed, further supporting this observation.

#### 4.2.3. Various Loads and Longitudinal Displacement

**Figure 19:**
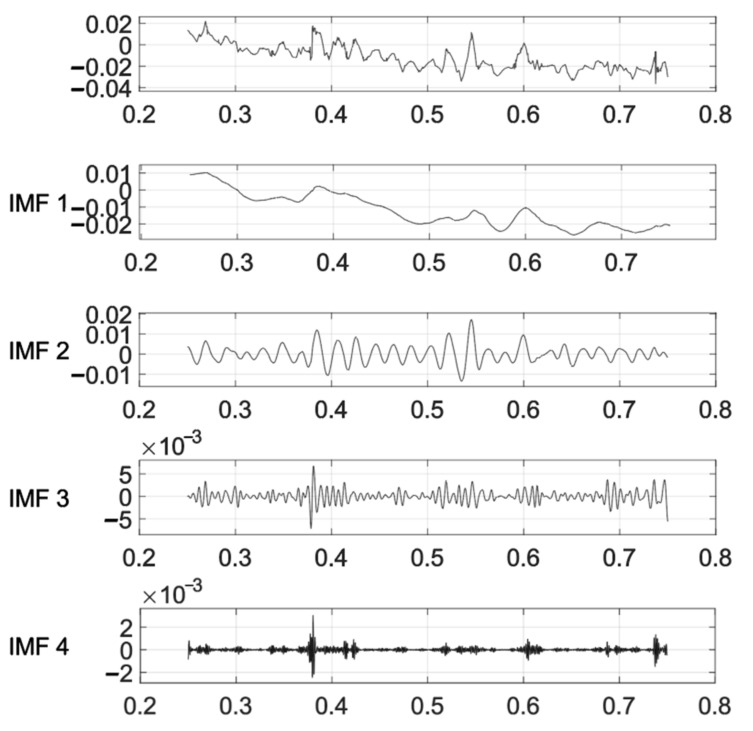
VMD of longitudinal displacement.

The longitudinal displacement of the bridge is influenced by multiple factors. In practical analysis, it is necessary to consider these factors comprehensively and apply appropriate compensation and treatment based on specific circumstances. In this study, the wavelet denoising and VMD methods were employed to analyze the longitudinal displacement data. In Figure 19, the results of the decomposed first four components, IMF1, IMF2, IMF3, and IMF4, are presented. These components represent different frequency components, with the low-frequency component often associated with long-term trends and low-frequency vibrations. In the longitudinal displacement analysis, IMF1 and IMF2 were selected, and their correlation coefficients with other parameters were calculated, respectively.

According to Table 3, the effects of air pressure, temperature, and Z-direction wind on longitudinal displacement generally show a positive correlation. Conversely, X- and Y-direction wind and humidity tend to demonstrate a negative correlation with longitudinal displacement. These observations are consistent with the patterns identified in the PDPs. Among these factors, the absolute values of the correlation coefficients between wind and humidity in the X and Z directions and the displacement IMF1 exceed 0.5. Meanwhile, they are relatively smaller between wind, air pressure, and temperature in the Y direction and the displacement IMF1. A possible reason for the discrepancy with the PDP results is that during the signal denoising process, noise components that may have similar effects are removed. According to the overall result of the calculated correlation coefficient, it can be judged that the longitudinal displacement is affected by multiple factors.

## 5. Conclusions

This paper proposes an approach that integrates a machine learning model and signal decomposition technique to globally and individually quantify the impact of environmental loads on the 3D displacements of the bridge and conducts in-depth research and validation on correlation analysis of the displacement components. The following conclusions can be drawn from the application of data from the long-span suspension bridge:

1. The correlation coefficient between vertical displacement and temperature of the bridge is significantly larger than that in the lateral and longitudinal directions. The reason is significantly related to the thermal expansion and contraction of the suspension bridge hanger and main cable.
2. Feature importance and PDP analysis based on the XGBoost displacement prediction model indicate that atmospheric pressure, Y-direction wind, and temperature have the highest importance scores for the displacements in the X, Y, and Z directions, respectively. The X-direction displacement gradually decreases as the atmospheric pressure increases, the Y-direction displacement shows a significant positive correlation with the wind speed, and the Z-direction displacement shows a significant negative correlation with the temperature.
3. The correlation analysis reveals that lateral deformation predominantly arises from lateral wind, while vertical deformation primarily results from temperature fluctuations. Longitudinal deformation is influenced by a combination of environmental factors. Specifically, it exhibits positive correlations with atmospheric pressure, temperature, and vertical wind, and has negative correlations with longitudinal wind, lateral wind, and humidity. These findings are consistent with the observations derived from the PDPs.

The method proposed in this paper is mainly based on the analysis of monitoring data from the bridge SHM system established on the Forth Road Bridge in Scotland and can be applied to other long-span bridges of different structures equipped with an SHM system. Since the GeoSHM system is currently not equipped with equipment to record vehicle loads, vehicle loads are not considered in this paper. In the future, we can consider acquiring more vehicle data and increasing the sensor types to gather a diversity of environmental loads.

## Figures and Tables

**Table 1:** The FRB restrictions under different wind speeds ^1^.

Wind Speed	Forth Road Bridge Restrictions
Gusts > 35 mph	40 mph speed limit on bridge
Gusts > 45 mph	Closed to double deck buses
Gusts > 50 mph	Closed to motorcycles, bicycles, and pedestrians
Gusts > 65 mph	Closed to all traffic

^1^ (https://www.theforthbridges.org/, accessed on 24 July 2023).

**Table 2:** Feature importance score.

	WindX	WindY	WindZ	Pressure	Humidity	Temperature
Bridge X	0.13	0.15	0.15	0.24	0.22	0.12
Bridge Y	0.05	0.91	0.01	0.01	0.01	0.01
Bridge Z	0.02	0.02	0.03	0.12	0.06	0.75

**Table 3:** Correlation coefficients between IMF1 and IMF2 and various environmental loads.

	Pressure	Temperature	WindX	WindY	WindZ	Humidity
IMF1	0.266	0.34793	−0.622	0	0.520	−0.514
IMF2	−0.084	−0.006	−0.134	−0.140	0.225	0.088

## Data Availability

Data are available on request due to restrictions, e.g., privacy or ethics. The data presented in this study are available on request from the corresponding author.
